# Impact of processing method on selected trace elements content of green tea: Does CTC green tea infusion possess risk towards human health?

**DOI:** 10.1016/j.fochx.2021.100173

**Published:** 2021-11-29

**Authors:** Himangshu Deka, Tupu Barman, Podma Pollov Sarmah, Arundhuti Devi, Pradip Tamuly, Tanmoy Karak

**Affiliations:** a Biochemistry Department, Tocklai Tea Research Institute, Jorhat 785008, Assam, India; b Analytical Services Department, Tocklai Tea Research Institute, Jorhat 785008, Assam, India; c Resource Management and Environment Section, Institute of Advanced Study in Science and Technology, Guwahati 781035, Assam, India; d Upper Assam Advisory Centre, Tea Research Association, Dikom 786101, Assam, India

**Keywords:** AAS, Atomic absorption spectrometer, ANOVA, Analysis of variance, BDL, Below detectable limit, CTC, Crush, tear and curl, CGT, CTC green tea, CGTI, CTC green tea infusion, CRM, Certified reference material, EDI, Estimated daily intake, FBD, Fluidized bed dryer, FSSAI, Food safety and standard authority of India, HCA, Hierarchical cluster analysis, HI, Hazard index, HQ, Hazard quotient, IDL, Instrument detection limit, MANOVA, Multivariate analysis of variance, OGT, Orthodox green tea, OGTI, Orthodox green tea infusion, PC, Principal component, PCA, Principal component analysis, PTDI, Provisional tolerable daily intake, RfD, Reference dose, WHO, World Health Organization, Cadmium (PubChem CID: 23973), Chromium (PubChem CID: 23976), Copper (PubChem CID: 23978), Iron (PubChem CID: 23925), Lead (PubChem CID: 5352425), Nickel (PubChem CID: 935), Zinc (PubChem CID: 23994), Orthodox green tea, CTC green tea, Infusion, Trace elements, Health hazard

## Abstract

This study reported the content of selected metals, viz. cadmium (Cd), chromium (Cr), copper (Cu), iron (Fe), nickel (Ni), lead (Pb) and zinc (Zn) as well as non-carcinogenic risks of orthodox green tea and CTC (crush, tear and curl) green tea (*Camellia sinensis* L.) in India. Results revealed that significantly higher amount of Cr (1.26–10.48 mg kg^−1^), Cu (13.40–22.73 mg kg^−1^), Fe (54.14–99.65 mg kg^−1^), Ni (3.43–7.09 mg kg^−1^), and Zn (25.04–38.04 mg kg^−1^) in CTC green tea than orthodox one. However, no definite trend was observed for Cd and Pb, with overall contents ranged from 6.68 to 23.32 µg kg^−1^ and 0.04 to 0.13 mg kg^−1^, respectively. The extraction of the elements in tea infusion was higher for CTC green tea. The hazard quotient and hazard index values of all the studied metals were less than unity, confirming no significant health effect for consumers assuming drinking of 750 mL tea infusion prepared from 10 g green tea per day per person.

## Highlights

• Reported Cd, Cr, Cu, Fe, Ni, Pb, and Zn contents in orthodox and CTC green teas.
• CTC green tea had a higher content of Cr, Cu, Fe, Ni, and Zn than orthodox one.
• Extraction of elements in CTC green tea was significantly higher than orthodox.
• In both types of tea hazard quotient and hazard index of analyte element were <1.

## Graphical abstract

**Figure:**
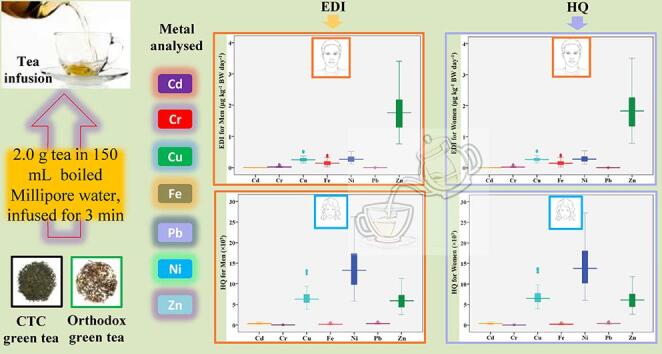

## Introduction

The environmental contaminants enter the human body through the food systems (Nkansah, Opoku, & Ackumey, 2016). Among the different contaminants, the accumulation of trace elements in the human body through the consumption of tea (*Camellia sinensis* L.) infusion (hot water extract of tea) has received global attention (Karak et al., 2017, Karak et al., 2017). The global consumption of tea is increasing over the years which stood at 5.53 million tonnes in 2016 (Food and Agriculture Organization of the United Nations, 2018). The acidic property of tea-growing soil makes the trace elements readily available for the plant to uptake (Karak and Bhagat, 2010, Bora et al., 2019). Recent literatures documented that the manufacturing processes of different types of tea from the young shoots of tea plant may contribute to the contents of a trace elements build up in made tea (Brzezicha-Cirocka et al., 2016, Szymczycha-Madeja et al., 2012, Zhang et al., 2018). Metals in the earth's crust coupled with anthropogenic activities ease the exposure of the several metals such as cadmium (Cd), chromium (Cr), copper (Cu), iron (Fe), lead (Pb), nickel (Ni), and zinc (Zn) into the environment. The major sources of soil contamination include agricultural inputs, viz. fertilizer and pesticides, industrial wastes, smelting and mining activities, vehicular emissions, etc. (Szymczycha-Madeja, Welna, & Pohl, 2015).

Made tea is processed from the young leaves comprising of young shoot and two leaves of the tea plant (*Camellia sinensis* L.). Based on processing conditions teas can be categorized into six varieties such as black, oolong, green, white, pu-erh and yellow tea (Deka, Sarmah, Devi, Tamuly, & Karak, 2021). Among these different types of tea, 75% black tea is consumed by the tea consumers followed by green tea (15%) which is the second most consumed tea in the world (Zhang et al., 2019). Processed tea (also known as made tea) is a vital source of different essential elements including trace elements and minerals for the human body. The content of trace elements in tea infusion is influenced by many factors such as type of tea, cultivar, geographical origin, extraction process, and elements content in tea. Karak, Paul, et al. (2017) reported that the regular drinking of tea serves as a dietary source for essential micronutrients like Cu, Fe and Zn. However, excessive accumulation and absorption of these elements may interfere with the physiological processes such as enzyme function leading to the development of stress inside the human body along with certain genetic disorders (Karak et al., 2017, Karak and Bhagat, 2010, Szymczycha-Madeja et al., 2012, Zhang et al., 2018). The contents of Cd, Cr, Pb and Ni in tea may be of serious concern for its consumers because of their carcinogenic nature (Zhong, Ren, & Zhao, 2016). The adverse health effects originating from Cd exposure include kidney damage, weakening of bone, etc. (Desideri, Meli, Roselli, & Feduzi, 2011). The hexavalent Cr (VI) is highly toxic and can cause harmful effects to the skin, kidney, liver and respiratory organs (Zhong et al., 2016). Lead can impair digestive and respiratory systems along with suppression of the immune system. Moreover, the nervous system and intelligence of children are severely affected by Pb exposure (Shen and Chen, 2008, Zhong et al., 2016). Excessive content of Ni in the human body can cause nickeleczema, a skin disorder (Zhong et al., 2016). Therefore, it is of utmost importance to determine the contents of trace elements in both tea and infusion as well as assessment of risk originating from tea consumption has been getting interest of the consumer.

Although the analysis of trace elements in black tea and its infusion from different parts of the world are dominantly available in the literatures, study on green tea and especially in Indian scenario is very limited. Moreover, elemental analysis of CTC green tea (CGT) and its comparison to orthodox green tea (OGT) has not been reported so far. As per the Food and Agriculture Organization of the United Nations, world green tea production is increasing at a much faster rate (5.4%) than that of black tea (3.0%) (Food and Agriculture Organization of the United Nations. 2018). Therefore, adequate attention needs to be paid towards green tea concerning manufacturing, quality and risk associated with consumption.

The potential health risk associated with the consumption of heavy metals through tea drinking can be assessed by hazard quotient (HQ) and the hazard index (HI). The HQ is a ratio of exposure to a particular chemical to its safe or maximum permissible exposure level. The HI is the sum of HQs for individual chemicals (USEPA, 2007). The HQ value of <1 for an element confirms no adverse effect on human health arising from its intake (USEPA, 2007). Several studies have been conducted to evaluate the impact of toxic metals from tea on human health (Barman et al., 2020, Karak et al., 2017, Li et al., 2015, Nkansah et al., 2016). Li et al. (2015) and Shen & Chen (2008) observed that HI values of trace elements from consumption of 1250 mL tea infusion per day prepared from 8 g tea were much lower than the safety limit.

In continuation of our earlier study on OGT and CGT, here we have reported the contents of non-toxic (Cu, Fe and Zn, also known as micronutrients) and toxic (Cd, Cr, Ni and Pb) elements in green tea and in their infusions. The biochemical quality parameters and sensory characteristics of OGT and CGT had been reported in our recently published work (Deka et al., 2020). Further, estimated daily intake (EDI), HQ and HI were also determined in the present study for health risk assessment upon consumption of green tea.

## Materials and methods

### Chemicals

Certified reference material (CRM) of Cu, Ni, Zn, Fe, Pb, Cd and Cr were procured from Sigma-Aldrich, India. Nitric acid (HNO_3_) and perchloric acid (HClO_4_) were obtained from Merck KGaA, Darmstadt, Germany.

### Tea leaves and processing of green tea

Young fresh shoots of cultivars TV1 (TV stands for Tocklai Vegetative), TV9, TV18, TV20, TV22, TV23 and TV25, RR17/144, Happy Valley 39, 482/12 and Ging186 grown in an identical environment and soil nutrient management practice were collected and green tea was processed in both orthodox and CTC mode following the protocol described by Deka et al. (2020).

The fresh samples were subjected to hot air blow in fluidized bed dryer (FBD) set at 100 ± 5 °C for 6 min for enzyme deactivation. The deactivated samples were allowed to cool immediately with the exposure of cold air followed by hand-rolling for 30 min. The mass samples were divided into two parts. One part of the rolled samples was subjected to 3 cut CTC and then dried in the FBD at 90 ± 2 °C for 20 min to have the CTC green tea. The other part of the rolled leaves was dried under identical conditions to have the orthodox green tea.

### Preparation of tea infusion

Tea infusion was prepared by brewing 2.0 g tea in 150 mL boiled millipore (Millipore Milli-Q Synthesis, Merck, Germany) water (Deka et al., 2020). The extracts were filtered after 3 min using Whatman no. 42 filter paper and stored in polypropylene screw-capped bottles for elemental analysis.

### Determination of metal content in green tea sample

#### Sample preparation

1.5 g homogenized tea sample was digested using HNO_3_ (17 mL) and HClO_4_ (4 mL) in a 250 mL conical flask. The mixture was evaporated until a clear solution was obtained. The mixture was allowed to cool down and transferred to a 25 mL volumetric flask through a Whatman no. 42 filter paper. The volume was made up with millipore water and stored in a polypropylene screw-capped bottle for analysis.

#### Working standards and calibration curve

**Fig. 1:**
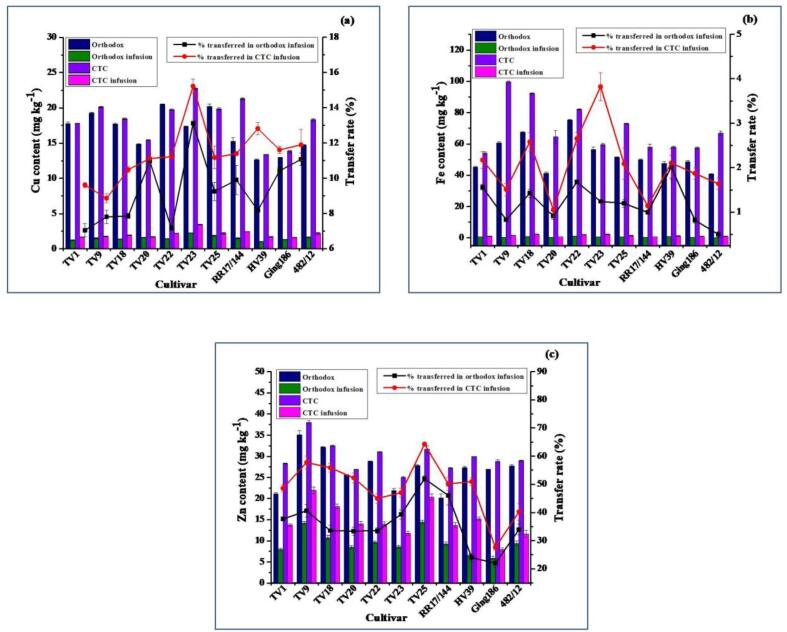
Average content (mg kg^−1^) of copper (Cu) (a), iron (Fe) (b), and zinc (Zn) (c) in orthodox and CTC green tea, in infusion, and their percent transfer into infusions (error bars specify the standard error). (For interpretation of the references to colour in this figure legend, the reader is referred to the web version of this article.)

Working standards of each element were prepared from the CRMs of concentration 1000 mg L^−1^ by diluting with 0.2% HNO_3_ solution. Calibration curves were prepared using the absorbance of each metal against concentration in µg or mg metal L^−1^ (Fig. 1S, Supplementary information). The R^2^ values of calibration curves ranged from 0.992 to 0.999.

#### Detection limit

The instrument detection limit (IDL) for each metal was estimated separately using the Association of Official Analytical Chemists method 971.20 (Latimer, 2019). In brief, the standard deviation of 20 blank determinations (n = 20) was multiplied by 3, using the standard flame condition and furnace program. The detection limit of the sample was estimated by multiplying the IDL with the dilution factor (Table 1S, Supplementary information).

#### Instrumentation

The contents of Cr, Cu, Fe, Ni and Zn in tea were determined using flame atomic absorption spectrometer (AAS) whereas Pb and Cd were determined using graphite furnace AAS (PerkinElmer, PinAAcle 900H). In infusion, the content of Fe and Zn were determined using flame AAS and the rests were determined using graphite furnace. The pre-specified flame and furnace programs will be found in the supplementary information (Tables 2AS, 2BS, 3AS-3ES).

#### Recovery

One tea sample was considered as control. This tea sample was analyzed seven times for determining the concentration of each element in question. The control sample was fortified at three different concentration levels of each element and then again analyzed using the procedure described in Sections “Sample preparation” and “Instrumentation”. Results showed satisfactory accuracy with recoveries ranging from 90.2 to 107.0 % (Table 4S, Supplementary information).

### Risk assessment

The EDI of metals on consuming five cups (10 g) of green tea in microgram per kilogram body weight (µg kg^−1^BW) per day was calculated by using the following formula as described in Deka et al. (2020)

(1) $$\mathrm{EDI}=\frac{C\times M\times T}{\mathrm{BW}\times 100}$$

where C is the mean concentration of the element in question in green tea; M is the weight of green tea consumed per day (10 g); T is the transfer rate in percentage and BW is the average body weight for men (67.4 kg) and women (64.9 kg).

The risk associated with the consumption of green tea infusions with different metal contents was assessed by determining the HQ. The HQ is calculated by using the following formula:

(2) $$\mathrm{HQ}=\frac{\mathrm{EDI}}{\mathrm{RfD}}$$

where RfD is the reference dose of the element in concern. The RfD values for Cd, Cr(III), Cu, Fe, Ni and Zn were 1.0 µg kg^−1^BW day^−1^ (Cao et al., 2010, Li et al., 2015), 1500 µg kg^−1^BW day^−1^ (Barman et al., 2020), 40 µg kg^−1^BW day^−1^ (Cao et al., 2010, Karak et al., 2017), 700 µg kg^−1^BW day^−1^ (Karak, Paul, et al., 2017), 20.0 µg kg^−1^BW day^−1^ (Li et al., 2015), and 300 µg kg^−1^BW day^−1^ (Cao et al., 2010, Karak et al., 2017), respectively. In the case of Pb, the provisional tolerable daily intake (PTDI) value (3.6 µg kg^−1^BW day^−1^) was used for calculation of HQ as RfD for Pb is not available (Li et al., 2015).

The combined risk can be assessed by determining the HI. The HI is calculated by using the following formula:

(3) $$\mathrm{HI}=\sum_{i=1}^{n}{\mathrm{HQ}}_{i}$$

where ${\mathrm{HQ}}_{i}$ is the HQ value of element i.

### Data analysis

All the analyses were carried out in three replications and data were presented as the mean ± standard error (SE). SAS software (version 9.4, SAS Institute Inc., USA) was used for performing analysis of variance (ANOVA). Differences between means were calculated using Tukey’s multiple comparison test and were considered significant at *p* ≤ 0.05 and *p* ≤ 0.01. Multivariate techniques such as hierarchical cluster analysis (HCA) and principal component analysis (PCA) were applied to the data set. Dimensionality reduction technique, viz. PCA was applied to the data. PCA is a data reduction technique that aims to explain most of the variances in the data whilst reducing the number of variables to a few uncorrelated components.

## Results and discussion

### Trace elements in tea

The contents of trace elements in OGT and CGT (Fig. 1) were in the order of Fe (40.86–99.65 mg kg^−1^) > Zn (20.20–38.04 mg kg^−1^) > Cu (12.62–22.73 mg kg^−1^) > Ni (2.61–7.09 mg kg^−1^) > Cr (0.51–10.48 mg kg^−1^) > Pb (0.04–0.13 mg kg^−1^) > Cd (6.68–23.32 µg kg^−1^). This trend was similar to the findings of Szymczycha-Madeja et al. (2012) who described tea as a major source of essential micronutrients, viz. Cu, Fe and Zn with trace quantities of other metals such as Cd and Pb. Li et al. (2015) observed a similar trend of trace elements content in commercial Chinese green tea samples. They reported that Pb content in commercially available Chinese green tea samples was higher than that of Cr. With the increasing maturity of tea leaves the concentrations of Cu and Zn decrease whereas Pb concentration increases (Szymczycha-Madeja et al., 2012). Therefore, green tea is supposed to have a high content of Cu and Zn and low content of Pb as this type of tea is processed from young shoots containing the apical bud with the first two leaves. Zinc content in green tea is higher than the black and oolong tea (Fernández et al., 2002, Shen and Chen, 2008). Shen & Chen (2008) concluded that the mineral loss during the fermentation stage of black and oolong tea manufacturing justifies the higher level of Zn in green tea. It was observed that green tea processed with the CTC method had a higher level of Cr, Cu, Fe, Ni, Pb and Zn as compared to the orthodox method. The higher content of these metals in CGT can be associated with the processing techniques (Jin et al., 2008, Polechońska et al., 2015, Szymczycha-Madeja et al., 2012). The enrichment of these metals was probably caused by external leaching during the CTC step of processing where the rollers used for the purpose were made of stainless steel (Polechońska et al., 2015, Zhang et al., 2018). This also corroborated the fact that the succus extracted from the tea leaves during the rolling and CTC stage gets contacted with the rollers of the CTC machine and this process increases the possibility of adhering the elements from the roller to the broken leaves (Polechońska et al., 2015, Zhang et al., 2018).

### Elements in tea infusion

**Fig. 2:**
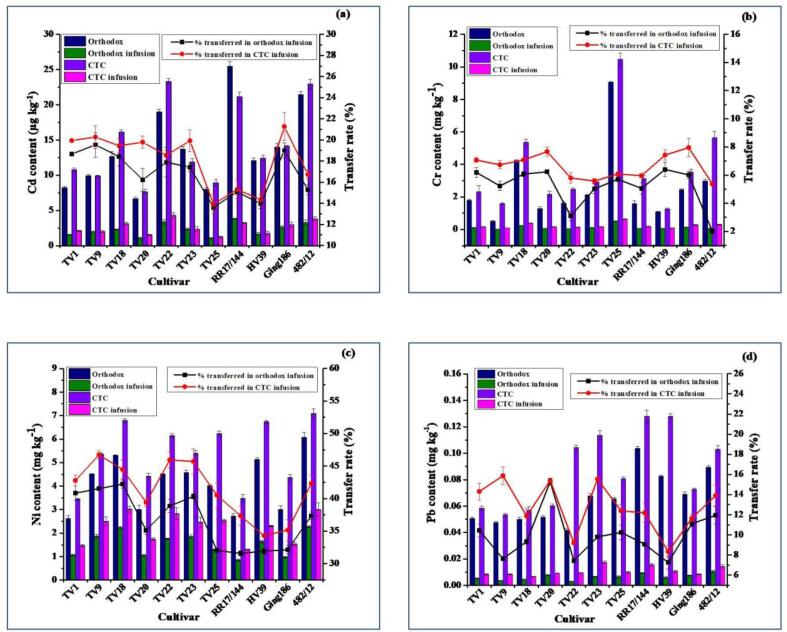
Average content of cadmium (Cd) (µg kg^−1^) (a), chromium (Cr) (mg kg^−1^) (b), nickel (Ni) (mg kg^−1^) (c) and lead (Pb) (mg kg^−1^) (d) in orthodox and CTC green tea, in infusion, and their percent transfer into infusions (error bars specify the standard error). (For interpretation of the references to colour in this figure legend, the reader is referred to the web version of this article.)

The trend in trace elements in tea infusions prepared from the two types of tea was significantly varied (Fig. 2). The general trend of analysed elements in tea infusions of the two types of tea were in the order Zn > Ni ∼ Cu > Fe > Cr > Pb > Cd. The release of trace elements from tea into its infusion mostly depend on the binding characteristics of these elements into the tea matrix as it reflects their solubility in water used for brewing (Polechońska et al., 2015). Trace elements in tea leaves form complexes with catechols, flavonols, tannins and polyphenols (Brzezicha-Cirocka et al., 2016, Szymczycha-Madeja et al., 2012). Numerous other factors, viz. water type, temperature, pH, duration of brewing, etc. also play a vital role in the variation in the extraction of the elements in tea infusion (Fernández et al., 2002, Szymczycha-Madeja et al., 2012). Temperature above 60 °C tends to reduce the extraction efficiency of Cu and Fe due to the insolubility of the polyphenolic complexes formed by these elements. The concentration of elements in tea infusion increases with time, however, the extraction rate for most of the elements is highest during the first five minutes of the brewing (Szymczycha-Madeja et al., 2012). CGT exhibited higher transfer rates of trace elements into infusion during brewing which could be attributed to the large surface area of made tea resulting from the CTC step involved during the processing (Deka et al., 2020, Liu et al., 2018, Polechońska et al., 2015).

### Copper content

The contents of Cu in OGT and CGT, their infusions and transfer rates from tea to its infusion are presented in Fig. 1(a). The Cu contents in OGT were in the range between 12.62 mg kg^−1^ in HV39 and 20.51 mg kg^−1^ in TV22 with a mean of 16.63 mg kg^−1^. The highest content in TV22 OGT was significantly different (*p* ≤ 0.01) from all other cultivars except TV25. In the case of CGT, the highest content was observed for TV23 (22.73 mg kg^−1^) which was significantly different (*p* ≤ 0.01) from all other CGTs, whereas HV39 (13.40 mg kg^−1^) had the lowest Cu level. The mean Cu content in CGT was 18.26 mg kg^−1^. The present findings of Cu content conform the reported data available in literatures (Han et al., 2005, Jin et al., 2008, Li et al., 2015). The average Cu content in 547 green tea samples of China was reported as 15.68 mg kg^−1^ (Han et al., 2005). Copper content in green tea available at the market of China and Poland ranged from 12.8 to 17.04 mg kg^−1^ (Jin et al., 2008, Li et al., 2015, Szymczycha-Madeja et al., 2015). However, comparatively higher amount of Cu (17.01–63.07 mg kg^−1^) were reported by Zhong et al. (2016) when several green tea available in China were documented. Copper content in tea leaves as well as made tea are positively correlated with the available Cu present in the soil where the plant grows. Contents of Cu in made tea was positively associated with soil pH and organic matter (Szymczycha-Madeja et al., 2012). In addition to that, the application of Cu-bearing bordeaux mixture, copper oxychloride to control plant disease and the use of Cu containing machinery for manufacturing of tea might be accountable for Cu concentration in made tea (Zhong et al., 2016, Tea Board of India, 2018). Food safety has been a global major concern in recent decades. Various countries and global bodies have formulated maximum permissible levels and put them into effect. In this study, the Cu contents in all samples were within the permissible limits set by different countries such as India (150 mg kg^−1^; FSSAI, 2020), China (60 mg kg^−1^; J. Zhang, Zhang, et al., 2018); Kenya (30 mg kg^−1^; Karak, Kutu, et al., 2017).

The extraction of trace elements into infusion was also significantly influenced by the processing methods. Notwithstanding, irrespective of tea types, Cu is extracted at a moderate rate when tea infusion is prepared (Szymczycha-Madeja et al., 2012). The contents of Cu in infusions were in the range from 1.03 to 2.27 mg kg^−1^ (equivalent to 0.05–0.11 mg L^−1^) in orthodox green tea infusions (OGTIs) and from 1.60 to 2.43 mg kg^−1^ (equivalent to 0.08–0.12 mg L^−1^) in CTC green tea infusions (CGTIs) with respective average transfer rates of 9.34% and 11.40%. However, higher extraction of Cu (24.4–27.0%) from green tea to its infusion has been documented by Brzezicha-Cirocka et al. (2016).

### Iron content

The contents of Fe in both types of green tea, their infusions and transfer rates are presented in Fig. 1(b). Iron contents were in the range from 40.86 mg kg^−1^ in cultivar 482/12 to 75.25 mg kg^−1^ in TV22 for OGT with the mean of 53.19 mg kg^−1^ and from 54.14 mg kg^−1^ in TV1 to 99.65 mg kg^−1^ in TV9 for CGT with the mean of 69.62 mg kg^−1^. Iron content in TV22 OGT was significantly different (*p* ≤ 0.01) from all other OGTs. In the case of CGTs, TV9 and TV18 had similar levels of Fe which were significantly different (*p* ≤ 0.01) from other CGTs. The Fe content in tea leaves is affected by factors such as growing soil, manufacturing technique, etc. (Zhang, Zhang, et al., 2018). Islam and Ebihara (2017) reported Fe content in Japanese green tea in the range from 57.8 to 105 mg kg^−1^ which confirms the present findings. Green tea originating from China, Sri Lanka, Japan, Nepal and South Korea had Fe content levels (40.7–116.0 mg kg^−1^) similar to the present study, however, Indian origin green teas were reported to have higher Fe content (146–195 mg kg^−1^) as reported by Koch et al. (2018). Higher levels of Fe (98.2–601 mg kg^−1^) in green tea were documented in other studies (Brzezicha-Cirocka et al., 2016, Fernández-Cáceres et al., 2001, Szymczycha-Madeja et al., 2015). In this study, it has been observed that the extraction of Fe from tea to its infusion during brewing is considered to be very poor which supports the findings of Szymczycha-Madeja et al. (2012). The transfer rates of Fe into infusions in the present study were in the range from 0.50 to 3.82% with CTC mode had an edge over the orthodox. This transfer rate is in agreement with that reported by Memić, Mahić, Žero, and Muhić-Šarac (2014) in the range from 1.35 to 1.90%. A higher level of extraction (10.9–13.4%) as compared to the present study was reported by Brzezicha-Cirocka et al. (2016) and Shen & Chen(2008). The contents of Fe in the infusions were in the range between 0.20 and 1.26 mg kg^−1^ (equivalent to 0.01–0.063 mg L^−1^) for OGTIs and between 0.66 and 2.38 mg kg^−1^ (equivalent to 0.033–0.119 mg L^−1^) for CGTIs. The Fe content in tea infusions from this study conforms with several earlier literatures (Fernández et al., 2002, Reto et al., 2007, Shen and Chen, 2008) which reported Fe content below 1 mg L^−1^. The low water extraction of Fe from made tea can be attributed to the presence of lower soluble Fe-polyphenols complex in tea (Brzezicha-Cirocka et al., 2016, Reto et al., 2007). The deposition of iron salts at pH above 4 might also contribute to the low extraction of Fe (Memić et al., 2014). To date, no limit has been set for iron content in tea infusion. However, in this study iron contents in all tea infusions were below the acceptable limit of 0.3 mg L^−1^ for drinking water as defined by the Bureau of Indian standard (BIS, 2012) and World Health Organization (WHO, 2011).

### Zinc content

Fig. 1(c) depicted the contents of Zn in both types of green tea, their infusions and transfer rates. Zn contents in OGT varied from 20.20 mg kg^−1^ (RR17/144) to 35.10 mg kg^−1^ (TV9) with a mean of 26.79 mg kg^−1^. The same in CGT varied from 25.04 mg kg^−1^ in (TV23) to 38.04 mg kg^−1^ in (TV9) with a mean of 29.87 mg kg^−1^. The Zn contents in TV9 for both OGT and CGT were significantly different (*p* ≤ 0.01) from other cultivars. These results of Zn content are in accordance with commercially available green tea in Bosnia and Herzegovina (21.8–30.8 mg kg^−1^) (Memić et al., 2014), Poland (25.6–46.1 mg kg^−1^) (Koch et al., 2018), South China (37.38 mg kg^−1^) (Zheng, Li, Li, Hu, & Li, 2014) and Italy (22.7–33.5 mg kg^−1^) (Desideri et al., 2011). Brzezicha-Cirocka et al. (2016) reported that Zn content in Indian green teas in the range from 38.1 to 38.7 mg kg^−1^. Another study covering major green tea producing countries like China and Japan documented a wide range (18.7–51.0 mg kg^−1^) of Zn content (Fernández-Cáceres et al., 2001). The Zn contents in all green tea samples were lower than the limit set by International Turkish Standard (50 mg kg^−1^); Brazilian Ministry of Health (250 mg kg^−1^); Australian Legal Requirements (750 mg kg^−1^); Ministry of Public Health, Thailand (667 mg kg^−1^); and WHO (200–500 mg kg^−1^) (Heidari, Bakhtiari, & Shirneshan, 2013). Among the studied elements, the average transfer of Zn into infusion was observed highest which were 35.99% for OGT and 49.07% for CGT. These results are in agreement with Brzezicha-Cirocka et al. (2016) and Memić et al. (2014) where they found that the transfer rates of Zn were 34.4–46.3% and 31.46–44.45%, respectively. Zn contents in infusions ranged from 5.92 mg kg^−1^ in Ging186 to 14.46 mg kg^−1^ in TV25 (equivalent to 0.30–0.72 mg L^−1^) for OGTIs and from 7.94 mg kg^−1^ in Ging186 to 21.98 mg kg^−1^ in TV9 (equivalent to 0.40–1.09 mg L^−1^) for CGTIs. In conformity with the present study, most literature reported Zn concentration in green tea infusion with below 1 mg L^−1^ (Fernández et al., 2002, Shen and Chen, 2008). Similar to our results, tea infusions prepared from green tea available in Sarajevo (Bosnia and Herzegovina) had a Zn content of 9.69 mg kg^−1^ (Memić et al., 2014).

### Cadmium content

The contents of Cd in both types of green tea, their infusions and transfer rates has been figured out in Fig. 2(a). Cadmium contents in OGT and CGT were in the range from 6.68 µg kg^−1^ in TV20 to 25.49 µg kg^−1^ in RR17/144 with a mean of 13.76 µg kg^−1^ and from 7.68 µg kg^−1^ in TV20 to 23.32 µg kg^−1^ in TV22 with a mean of 14.49 µg kg^−1^, respectively. OGT processed from cultivar RR17/144 had significantly higher (*p* ≤ 0.01) content of Cd. CGT of TV22 and 482/12 had similar levels of Cd content which were significantly higher (*p* ≤ 0.05) in comparison to other CGTs. The Cd contents in all green teas were below the maximum acceptable limit set by Food safety and standard authority of India (FSSAI) (1500 µg kg^−1^; FSSAI, 2020); WHO (300 µg kg^−1^; WHO, 1998); Germany (200 µg kg^−1^), Vietnam (1000 µg kg^−1^; Koch et al., 2018). In the present study, Cd levels are much lower than available literatures (Brzezicha-Cirocka et al., 2016, Desideri et al., 2011, Han et al., 2005, Li et al., 2015, Lisia et al., 2015, Zheng et al., 2014) in which the authors reported the average Cd content in green tea ranged between 90 and 670 µg kg^−1^. The variations in Cd content might be due to different doses of phosphatic fertilizers to the soil and environment of the region where a particular tea cultivar has been grown. Ma et al. (2016) also detected lower levels of Cd in 56 Biluochun green teas in the range from 35.5 to 53.01 µg kg^−1^. Zhang and Fang (2007) observed that the Cd level in green tea leaves (12–57 µg kg^−1^) of China was lowest among different trace elements which are similar to our findings. The average Cd extraction efficiency of CGT (18.13%) was higher than that of OGT (16.82%) with respective concentrations ranging from 1.24 to 4.32 µg kg^−1^ (equivalent to 0.06–0.22 µg L^−1^) and from 1.08 to 3.84 µg kg^−1^ (equivalent to 0.05–0.19 µg L^−1^). The extraction of Cd in infusions in the present study conformed with Brzezicha-Cirocka et al. (2016) (9.41–43.8%) and de Oliveira et al. (2018) (5–21%). In agreement with our results, Li et al. (2015) detected Cd at a level of 0.05 µg L^−1^ in the infusions of 26 green teas from China.

### Chromium content

Fig. 2(b) represents the contents of Cr in the analyzed teas with their infusions and transfer rates. Cr contents ranged between 0.51 mg kg^−1^ in TV9 and 9.06 mg kg^−1^ in TV25 with a mean of 2.61 mg kg^−1^ for OGT, whereas it was between 1.26 mg kg^−1^ in HV39 and 10.48 mg kg^−1^ in TV25 with a mean of 3.72 mg kg^−1^ for CGT. Chromium content in both types of green teas processed from TV25 was significantly higher (*p* ≤ 0.01) as compared to other cultivars. In line with our results, Cr level in Chinese green tea was reported in a wide range from below detectable limit (BDL) to 16.10 mg kg^−1^ as reported by Han et al. (2005). The Cr contents in the present study conforms to the reported results by Brzezicha-Cirocka et al. (2016) (0.3–3.4 mg kg^−1^), de Oliveira et al. (2018) (0.41–4.60 mg kg^−1^), Desideri et al. (2011) (<2–2.5 mg kg^−1^) and Zheng et al. (2014) (0.49–2.22 mg kg^−1^). The extraction efficiency of Cr in infusions was observed at 5.18 and 6.61% for OGT and CGT, respectively. Recent literatures reported the higher extraction of Cr (12.4–79%) in infusion as compared to the present study indicating the lower risk of Cr toxicity arising from the consumption of green teas being reported in the current study (Brzezicha-Cirocka et al., 2016, de Oliveira et al., 2018, Shen and Chen, 2008). The Cr concentrations in OGTIs were in the range from 0.03 to 0.52 mg kg^−1^ (equivalent to 1.5–26 µg L^−1^) and in CGTIs it was from 0.09 to 0.64 mg kg^−1^ (equivalent to 4.5–32 µg L^−1^). The Cr contents in green tea infusions were less than the maximum permissible limit of 50.0 µg L^−1^ for drinking water set by WHO (WHO, 2011) and Bureau of Indian standard (BIS, 2012).

### Nickel content

The contents of Ni in both types of green tea, their infusions and transfer rates are presented in Fig. 2(c). Nickel content was highest in cultivar 482/12 (OGT: 6.06 mg kg^−1^, CGT: 7.09 mg kg^−1^) and lowest in TV1 (OGT: 2.61 mg kg^−1^, CGT: 3.43 mg kg^−1^) with a mean of 4.13 and 5.40 mg kg^−1^ for OGT and CGT, respectively. The Ni level in 482/12 OGT was significantly different (*p* ≤ 0.01) from other cultivars. CGT processed from 482/12, TV18 and HV39 had comparable Ni levels which were significantly higher (*p* ≤ 0.01) than other cultivars. Ni content in the present study is in agreement with previous literatures such as Desideri et al. (2011) (3.2–7.2 mg kg^−1^), Memić et al. (2014) (3.76–9.46 mg kg^−1^), Szymczycha-Madeja et al. (2015) (2.80–8.25 mg kg^−1^), and Brzezicha-Cirocka et al. (2016) (3.7–12.2 mg kg^−1^). Szymczycha-Madeja et al. (2012) reported that extraction of Ni is very high during tea brewing than other metals. In the present study, Ni extraction efficiency ranging from 31.6 to 46.7% was observed. It was found that CGTIs had 6% more Ni content when compared with OGTIs. A similar extraction rate was reported by Brzezicha-Cirocka et al. (2016) (34.2–38%) in 41 green tea samples from major tea producing countries. Ni concentrations in OGTIs were in the range between 0.86 mg kg^−1^ and 2.26 mg kg^−1^ (equivalent to 43–113 µg L^−1^), whereas in CGTIs it was between 1.30 mg kg^−1^ and 3.02 mg kg^−1^ (equivalent to 65–151 µg L^−1^). Comparatively lower amount of Ni content (17.69 µg L^−1^) in the infusions of 26 green teas from Jiangxi, China has been reported by Li et al. (2015). The variations of Ni content in infusions can be attributed to brewing method parameters such as temperature, duration, tea/water ratio as well as the solubility pattern of Ni-polyphenol complexes in made tea.

### Lead content

Fig. 2(d) depicted the contents of Pb in both types of green tea, their infusions and transfer rates. It has been observed that Pb content in OGT varied from 0.04 mg kg^−1^ in TV22 to 0.10 mg kg^−1^ in RR17/144 with a mean value of 0.07 mg kg^−1^. The Pb content in RR17/144 OGT was significantly different (*p* ≤ 0.01) from all other cultivars. CGT had higher Pb content with values ranging from 0.05 mg kg^−1^ (TV9) to 0.13 mg kg^−1^ (RR17/144 and HV39) with a mean value of 0.09 mg kg^−1^. The Pb content in CGTs of RR17/144 and HV39 was significantly different (*p* ≤ 0.01) from the remaining cultivars. Recent studies reported a similar trend of Pb contents in green tea available at super markets of Ghana (0.10–0.20 mg kg^−1^), Brazil (0.03–0.1542 mg kg^−1^) and Bosnia and Herzegovina (BDL-0.92 mg kg^−1^) (Lisia et al., 2015, Memić et al., 2014, Nkansah et al., 2016). Shen & Chen (2008) determined a mean Pb content of 0.01 mg kg^−1^ in 15 green tea samples from Taiwan. The Pb contents detected in the present study were lower than several previous literatures on green tea ranged between 0.12 and 4.3 mg kg^−1^ (Desideri et al., 2011, Szymczycha-Madeja et al., 2015, Koch et al., 2018, Li et al., 2015, de Oliveira et al., 2018, Ma et al., 2016, Zheng et al., 2014). Moreover, the Pb contents determined in the present study were lower than the maximum permissible limit of 10.0 mg kg^−1^ set by US Pharmacopeia, WHO (Nkansah et al., 2016). The maximum permissible limit of Pb in made tea for India has been decreased to 5 mg kg^−1^ from its earlier limit of 10 mg kg^−1^ (FSSAI, 2020). The Pb contents in these analyzed samples were found much lower than the concentration of Pb prescribed by the Indian authority (FSSAI, 2020). The wide variation in Pb concentration can be justified by contamination arising from different anthropogenic sources like Pb-bearing dust deposition on the surface of tea leaves (Brzezicha-Cirocka et al., 2016). Furthermore, the availability of Pb for tea plant uptake increases significantly with a decrease in soil pH. Therefore, the potential bioavailability of Pb contaminated tea growing soil may facilitates its uptake by root and its subsequent deposition in tea leaves (Zheng et al., 2014). The concentrations of Pb in infusions were higher for CGT (0.007–0.018 mg kg^−1^, equivalent to 0.35–0.90 µg L^−1^) as compared to orthodox counterpart (0.003–0.011 mg kg^−1^, equivalent to 0.15–0.55 µg L^−1^) with respective average transfer rates of 12.79 and 9.96%. The observed extraction of Pb in this study was in line with Shen & Chen (2008) and Brzezicha-Cirocka et al. (2016) where the authors reported that extraction of Pb ranges between 7.1 and 39.3%. On the other hand, the detected Pb contents in infusion for this present study were found to be higher than infusions prepared from commercial green teas of Taiwan (0.1 µg L^−1^) (Shen & Chen, 2008), but much lower than those in the infusions of green tea from China (2.80 µg L^−1^) (Li et al., 2015), USA and China (1.01–4.00 µg L^−1^) (de Oliveira et al., 2018) Brazil (<5–15 µg L^−1^) (Lisia et al., 2015). Moreover, the Pb levels in infusions were lower than the limit (10 µg L^−1^ for drinking water) set by WHO (WHO, 2011) and Bureau of Indian standard (BIS, 2012) and Standardization Administration of the People’s Republic of China (Li et al., 2015).

### Health risk analysis

#### Estimated daily intake (EDI) of trace elements

**Fig. 3:**
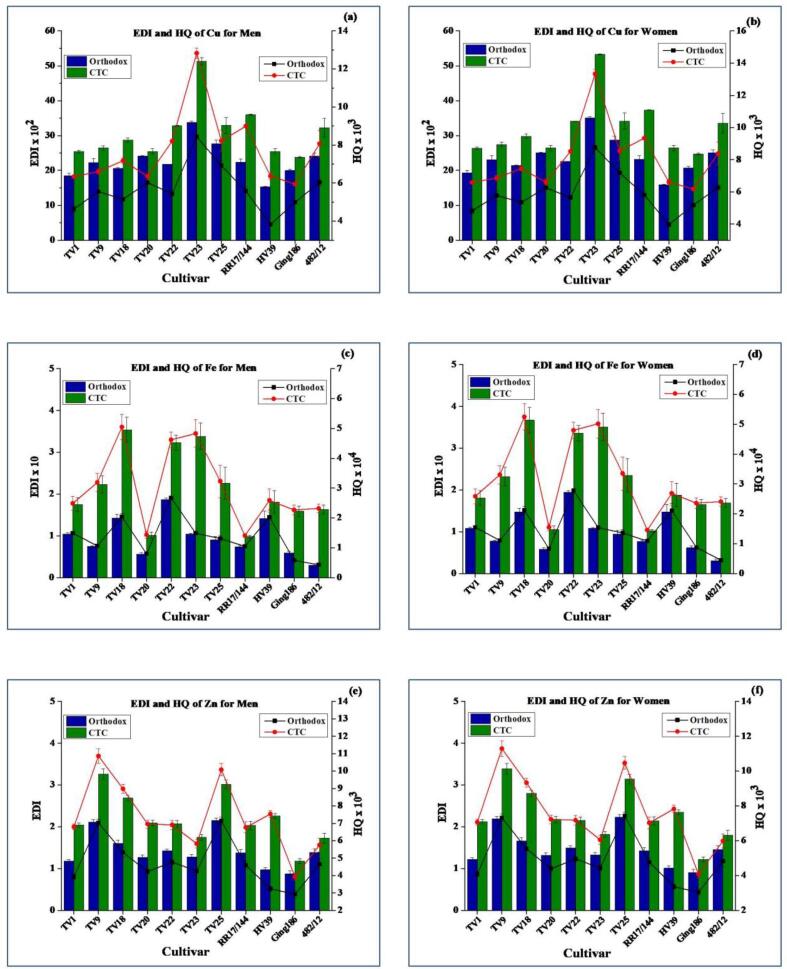
Estimated daily intake (EDI) and hazard quotient (HQ) of Cu (a: men; b: women), Fe (c: men; d: women), and Zn (e: men; f: women) (average body weight considered for men and women were 67.4 and 64.9 kg, respectively).

**Fig. 4:**
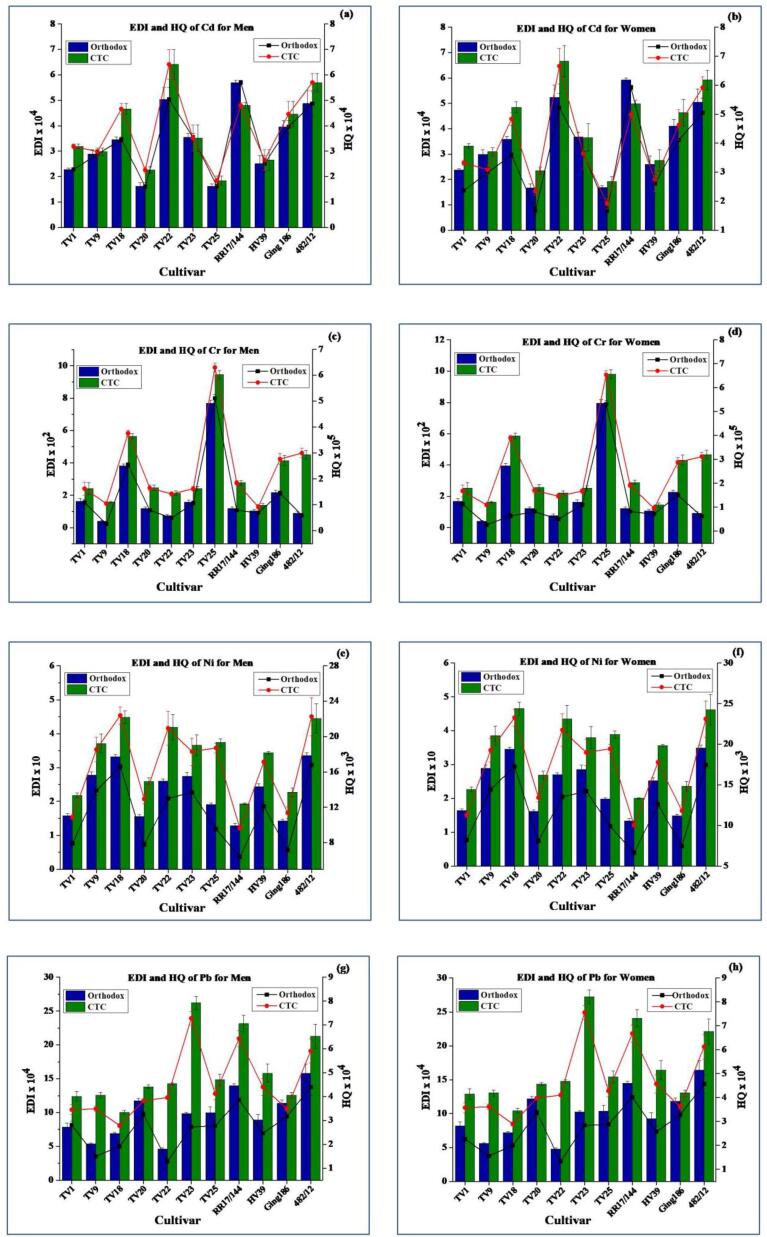
Estimated daily intake (EDI) and hazard quotient (HQ) of Cd (a: men; b: women), Cr (c: men; d: women), Ni (e: men; f: women), and Pb (g: men; h: women) (average body weight considered for men and women were 67.4 and 64.9 kg, respectively).

The EDI values of trace elements through consumption of tea are presented in Fig. 3, Fig. 4. The general trends of EDI values for OGT and CGT were in the order of Zn > Cu > Ni > Fe > Cr > Cd > Pb and Zn > Ni > Cu > Fe > Cr > Pb > Cd, respectively. The EDIs through consumption of CGT were higher than those for OGT owing to the higher extraction efficiency. The EDI values for OGT and CGT were in the range from 4.60 × 10^−4^ to 2.23 and 1.84 × 10^−4^ to 3.39 µg kg^−1^ bw day^−1^_,_ respectively. EDI values of the analyzed elements for both types of green tea were much lower than the corresponding RfD and PTDI (for Pb) values. Therefore, EDI values indicate that consumption of green teas does not seem to any adverse health hazard for consumers.

#### Hazard quotient (HQ)

HQ has been successfully tested by several published literatures on trace elements content of tea (Barman et al., 2020, Cao et al., 2010, Li et al., 2015, Nkansah et al., 2016, Shen and Chen, 2008). In this study, health risk was assessed by determining the HQ value through consumption of 10 g of tea per day (equivalent to 750 mL tea infusion). The HQ values are presented in Fig. 3, Fig. 4. All the HQ values (0.26 × 10^−5^–23.27 × 10^−3^) were far below 1 indicating that daily intake of 10 g green tea would not pose any health hazard. HQ values calculated for women were found slightly higher due to higher EDI values as well as lower body weight than men.

The mean HQ values resulting from the consumption of OGT were in the order of Ni > Cu > Zn > Cd > Pb > Fe > Cr. However, in CGT Pb had higher HQ values than Cd due to increased Pb content as well as higher extraction efficiency. In conformity with the present study, Li et al. (2015) reported comparable HQ values for Cu (3.54 × 10^−3^–5.68 × 10^−2^) and Cr (2.33 × 10^−5^–1.69 × 10^−4^). The HQ values for Cu were 10 times less than those of pu-erh tea (Cao et al., 2010). The HQ values for Pb were comparable with that reported for green tea (4.2 × 10^−4^) by Shen & Chen (2008). The present findings are in close agreement with the recent study by Barman et al. (2020) where the authors reported that HQ values for Cr in teas collected from Assam and North Bengal in the range from 3 × 10^−5^ to 8 × 10^−5^.

#### Hazard index (HI)

The HI values for both OGT and CGT (1.59 × 10^−2^−4.14 × 10^−2^) were far below 1 (Table 5S, Supplementary information), indicating no carcinogenic risk and other health hazards to humans arising from the consumption of these green tea infusions. The HI values determined in this study were much lower than pu-erh tea (17 × 10^−2^−29 × 10^−2^) from China. This could be due to the higher extraction efficiency of metals in pu-erh tea compared to other types of tea (Cao et al., 2010). Nkansah et al. (2016) reported HI values of 69 × 10^−2^ for three green teas available in Ghana. Shen & Chen (2008) observed a low HI value (1.3 × 10^−3^) when studied four elements (As, Cr, Cd, Pb) in 15 green tea samples. The authors further observed that the HI value for green tea was much lower than that of black tea (6.7 × 10^−1^) and oolong tea (2.4 × 10^−1^).

### Statistical interpretation

**Fig. 5:**
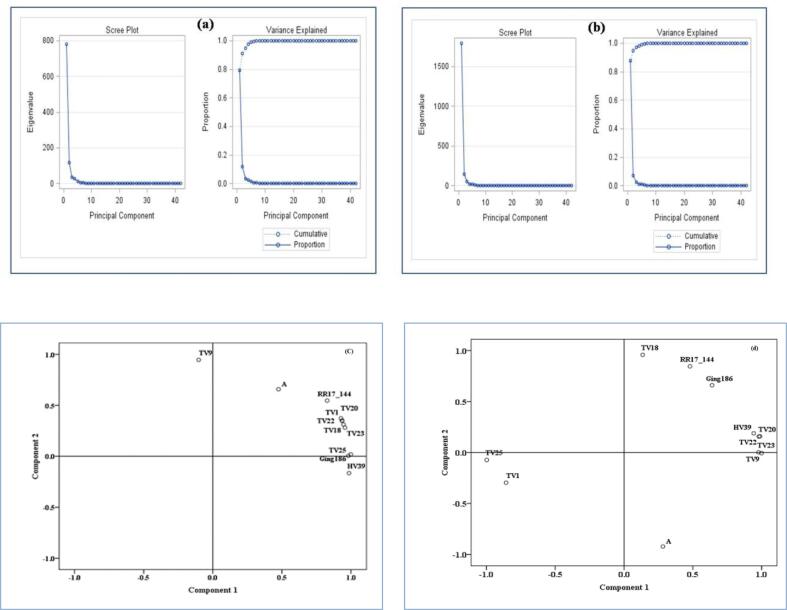
Scree plot and variance explained plot for orthodox (a) and CTC (b) green tea as well as loading plots obtained in PCA for orthodox (c) and CTC (d) green tea (in PCA plot ‘A’ stands for ‘482/12’ and ‘RR17_144’ stands for ‘RR17/144’). (For interpretation of the references to colour in this figure legend, the reader is referred to the web version of this article.)

PCA was performed on the obtained data in order to summarize the linear relationships among a set of response variables. The optimum number of principal components (PCs) was determined by the scree plot along with the cumulative variance explained by the PCs. It may be seen from the scree plot and variance explained plot (Fig. 5), that for both OGT and CGT, two PCs explained more than 90% of variability in the response variable. The first PC explained 79.39 and 87.85% of variability in OGT and CGT, respectively whereas the second PC explained 11.9 and 6.95% respective variability.

**Fig. 6:**
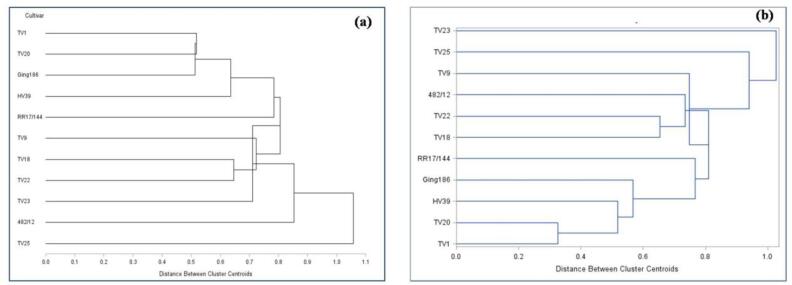
Dendrogram representing clustering of cultivars based on different parameters for orthodox (a) and CTC (b) green tea. (For interpretation of the references to colour in this figure legend, the reader is referred to the web version of this article.)

HCA was applied in order to identify the presence of homogenous groups among different cultivars based on all the investigated variables separately for two processing methods namely orthodox and CTC. In order to obtain homogenous groups, cluster analysis is performed as the most widely used technique for unsupervised pattern recognition in chemometrics. Similarly, hierarchical clustering of data was applied based on the core idea that nearby objects are more related than those who are far away. Prior to PCA and cluster analyses, the datasets were standardized to make the entire variables unit free. It may be seen from Fig. 6, that the cultivar TV25 and TV23 are distinctly different from remaining cultivars in orthodox and CTC mode, respectively.

All the variables were subjected for statistical analysis through multivariate analysis of variance (MANOVA), multivariate treatment contrast analysis and Wilk’s Lambda criterion to find out the best cultivar and also combination of cultivar and methods. MANOVA was applied separately for two different methods to find out overall significant differences among the cultivars. A perusal of Tables 6S and 7S (Supplementary information) indicated that with respect to Wilks' Lambda, Pillai's Trace, Hotelling-Lawley Trace, Roy's Greatest Root statistics, in both the methods i.e. orthodox and CTC, there was overall significant differences among the cultivars. The same conclusion could be drawn when the analysis was performed for both the methods taken together (Table 8S, Supplementary information). To investigate any significant difference between the two methods i.e. orthodox and CTC, MANOVA was carried out by taking care of all the response variables and it was found that the methods differ significantly (Table 9S, Supplementary information).

## Conclusion

This study highlighted the contents as well as compared the trace elements, viz. Cu, Fe, Zn, Ni, Cr, Pb and Cd in OGT and CGT processed from the same cultivars. The contents of Cr, Fe, Ni and Zn in CGT were higher. The CGTI had higher extraction of elements when compared to OGTI. However, the EDI values for all the analyzed elements in both types of tea are well within the safety limit of human consumption. Moreover, the HQ values for each element and the HI values for each cultivar are well below 1 which indicates that the consumption of these teas is free from the risk of any significant health hazard.

## CRediT authorship contribution statement

**Himangshu Deka:** Conceptualization, Methodology, Resources, Investigation, Formal analysis, Validation, Writing – original draft. **Tupu Barman:** Methodology, Investigation, Formal analysis, Validation, Writing – review & editing. **Podma Pollov Sarmah:** Visualization, Writing – review & editing. **Arundhuti Devi:** Supervision. **Pradip Tamuly:** Supervision. **Tanmoy Karak:** Data curation, Validation, Writing – review & editing.

## Declaration of Competing Interest

The authors declare that they have no known competing financial interests or personal relationships that could have appeared to influence the work reported in this paper.
