# Supplementary material for: Impact of processing method on selected trace elements content of green tea: Does CTC green tea infusion possess risk towards human health?
Source: Food Chem X. 2021 Nov 29;12:100173. doi: 10.1016/j.fochx.2021.100173 (PMC8645460; doi:10.1016/j.fochx.2021.100173)
Supplement: Supplementary data 1 [file mmc1.docx]

**Supplementary information**

**Impact of processing method on selected trace elements content of green tea: Does CTC green tea infusion possess risk towards human health?**

Himangshu Deka^a,*^, Tupu Barman^b^, Podma Pollov Sarmah^a^, Arundhuti Devi^c^, Pradip Tamuly^a^, Tanmoy Karak^d,*^

^a^Biochemistry Department, Tocklai Tea Research Institute, Jorhat 785008, Assam, India

^b^Analytical Services Department, Tocklai Tea Research Institute, Jorhat 785008, Assam, India

^c^Resource Management and Environment Section, Institute of Advanced Study in Science and Technology, Guwahati 781035, Assam, India

^d^Upper Assam Advisory Centre, Tea Research Association, Dikom 786101, Assam, India

^*^ Corresponding author. Tel.: +91-9864462965 (H. Deka); +91-9435861567 (T. Karak)

E-mail addresses: [himangshu1234@gmail.com](mailto:himangshu1234@gmail.com) (H. Deka)

[tanmay.karak@gmail.com](mailto:tanmay.karak@gmail.com); [t.karak@tocklai.net](mailto:t.karak@tocklai.net) (T. Karak)

**Calibration curves**


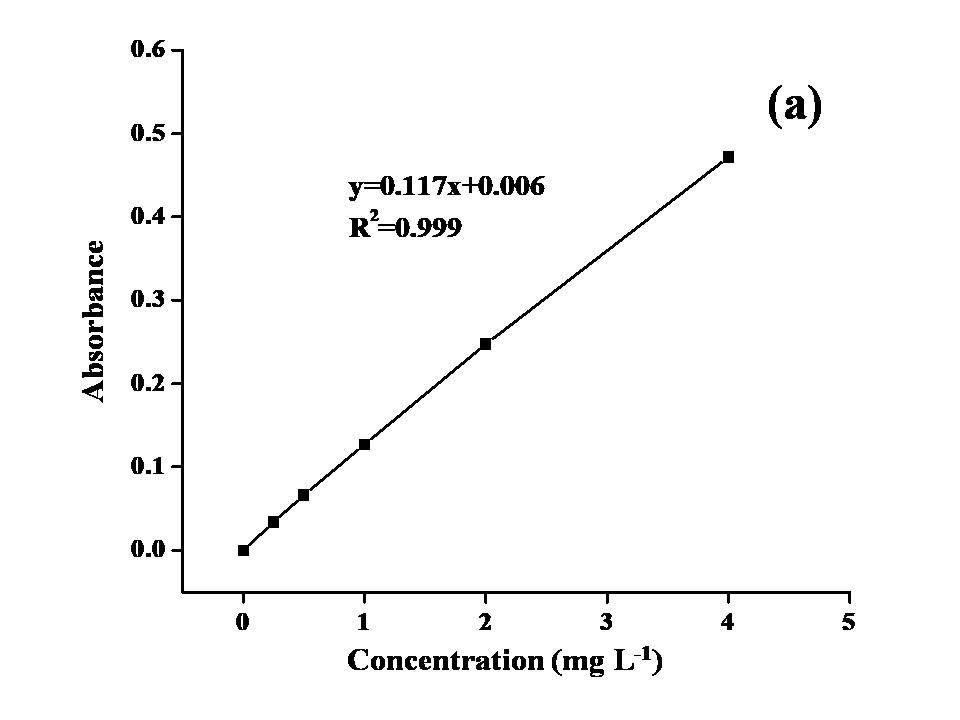

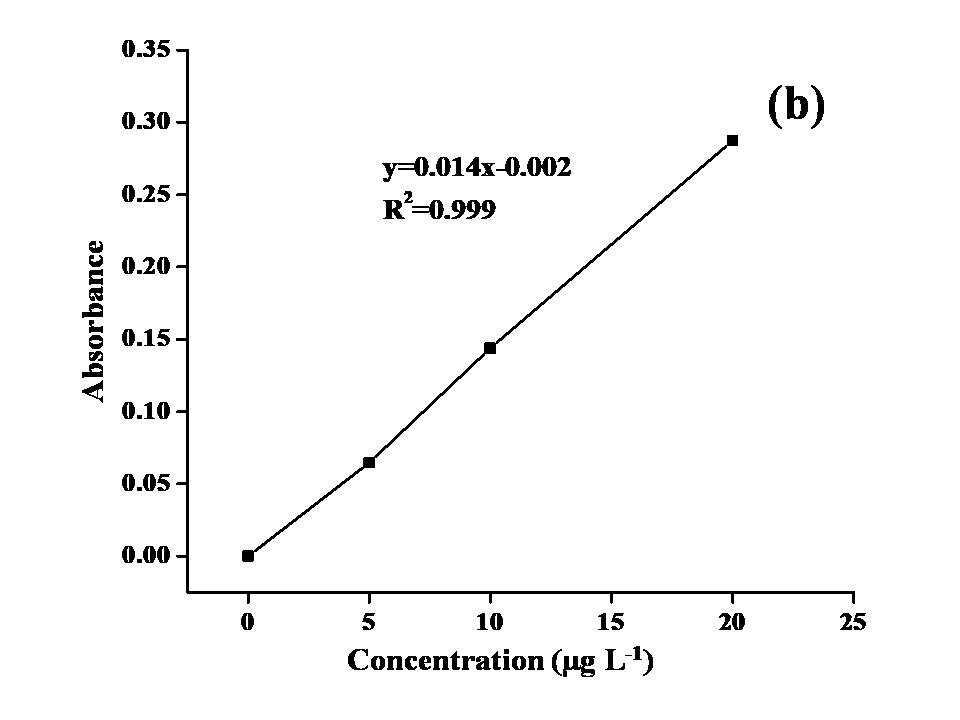


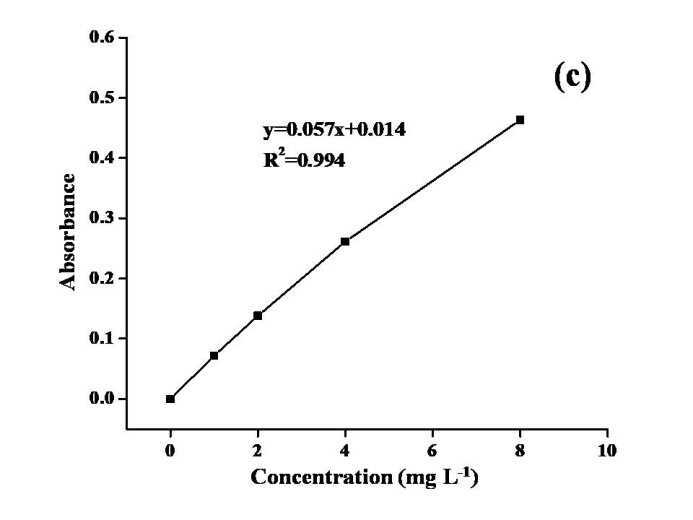

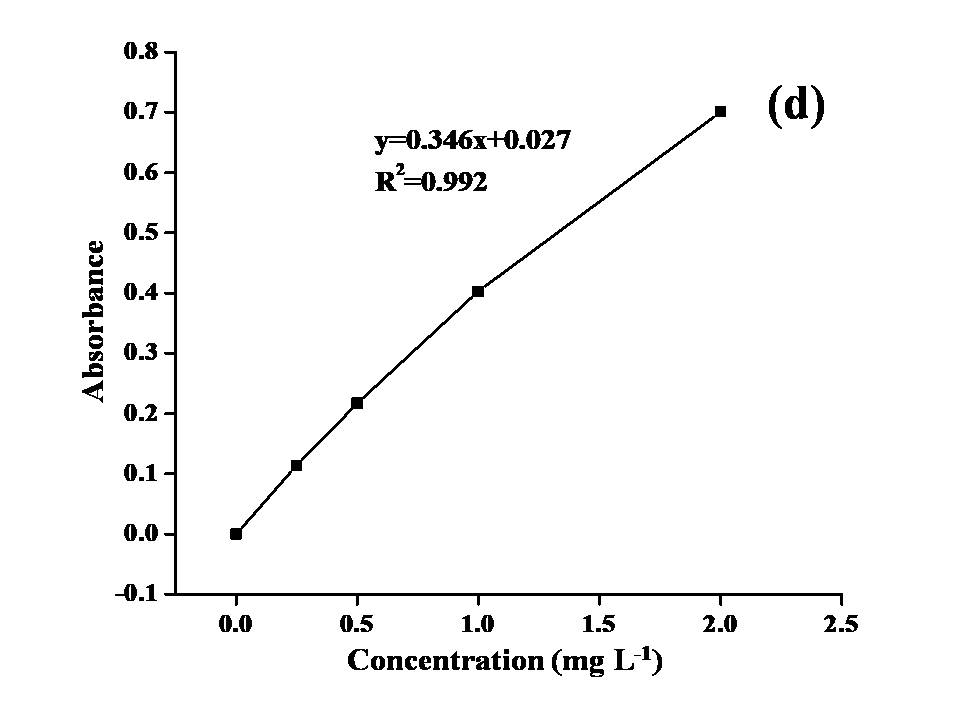


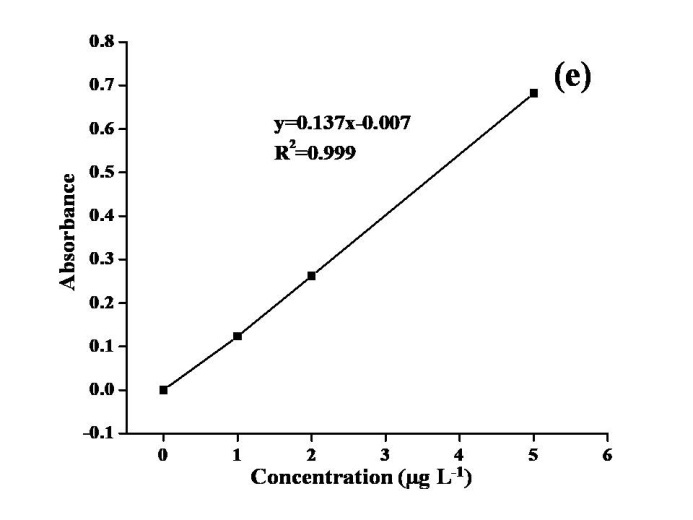

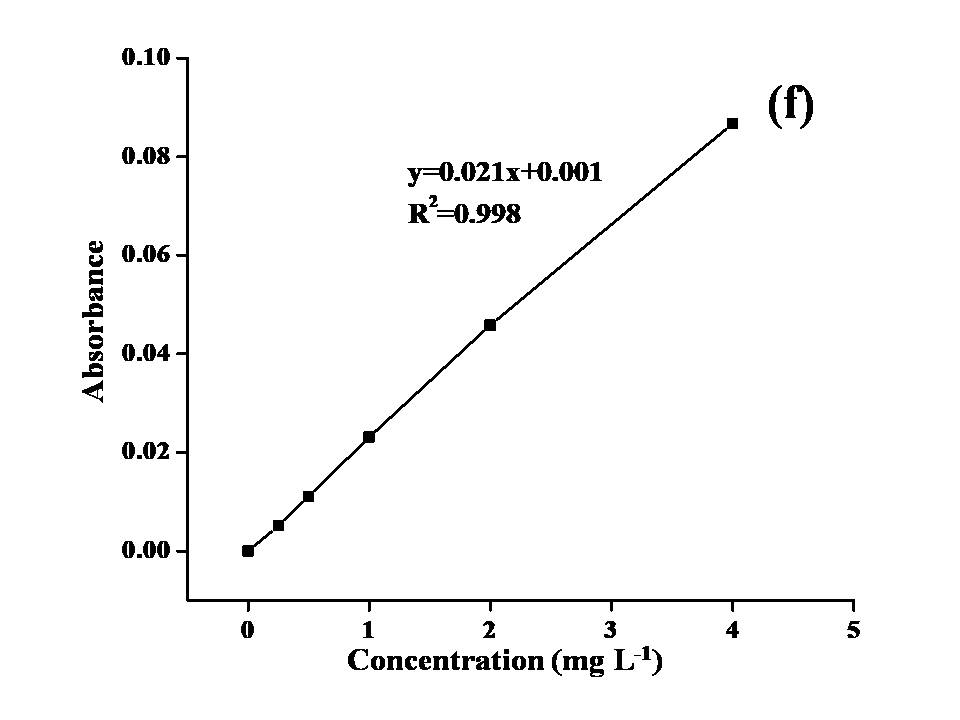


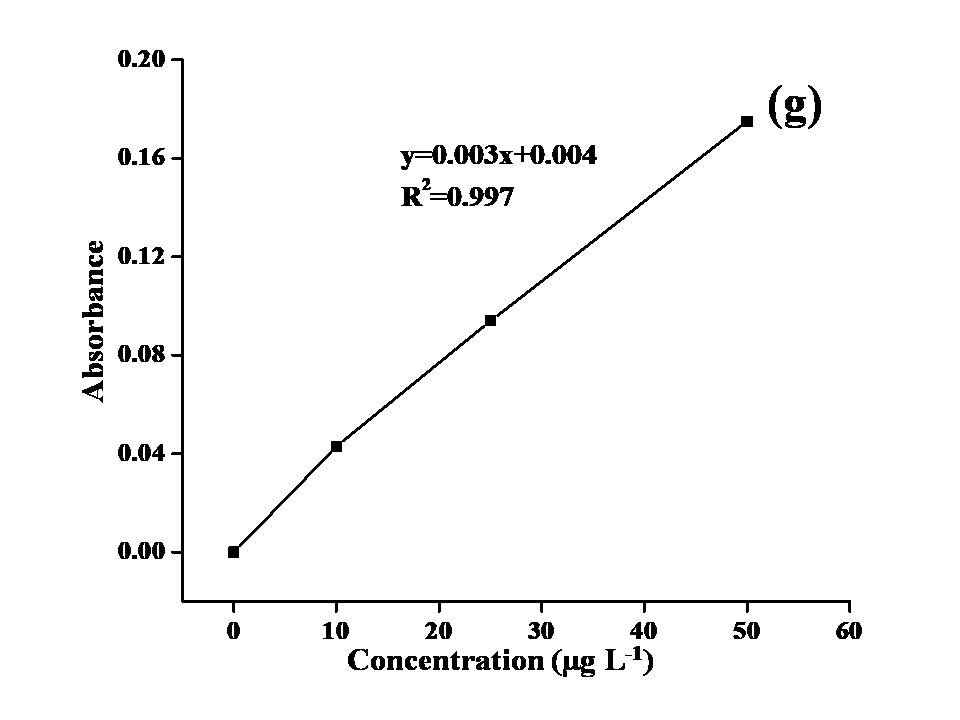

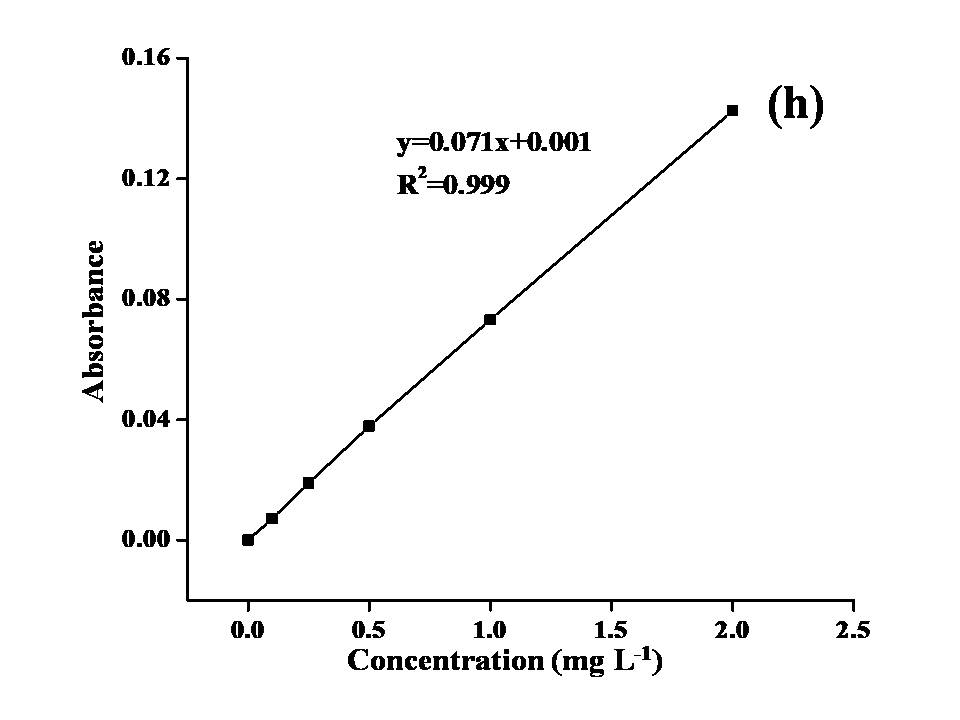


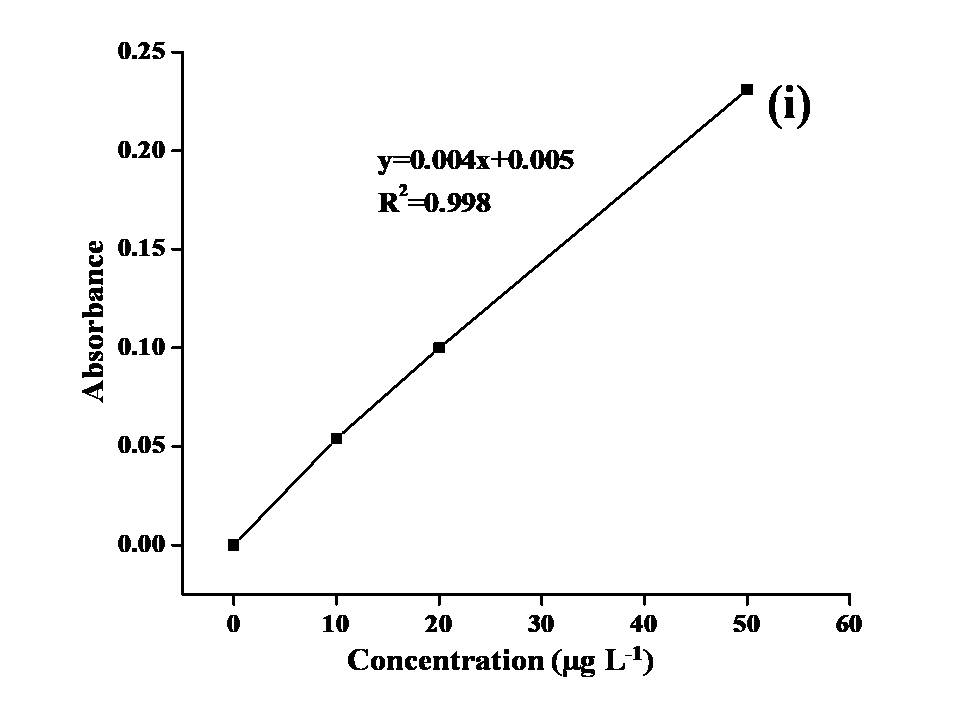

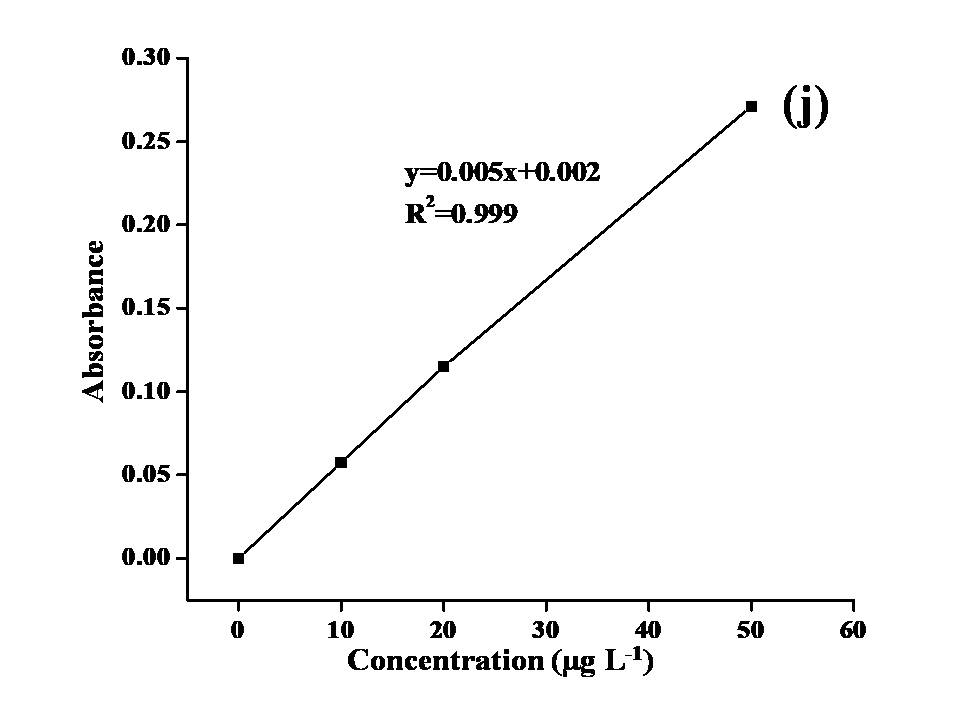


**Fig. 1S.**Standard calibration curves of (a) Cu in FAAS, (b) Cu in GFAAS, (c) Fe in FAAS, (d) Zn in FAAS, (e) Cd in GFAAS, (f) Cr in FAAS, (g) Cr in GFAAS, (h) Ni in FAAS, (i) Ni in GFAAS, and (j) Pb in GFAAS.

**Table 1S**

The estimated instrument detection limit (IDL)

| **Analyte** | **IDL (mg kg^-1^)** | |
| --- | --- | --- |
|  | **Flame** | **Furnace** |
| Copper | 0.05 | 0.001 |
| Chromium | 0.10 | 0.002 |
| Iron | 0.05 | --- |
| Nickel | 0.10 | 0.001 |
| Zinc | 0.025 | -- |
| Lead | -- | 0.001 |
| Cadmium | -- | 0.0001 |

**Table 2AS**

Operating parameters for flame atomic absorption spectrometer (FAAS)

| **Operating parameters** | **Analyte** | | | | |
| --- | --- | --- | --- | --- | --- |
|  | **Cu** | **Cr** | **Fe** | **Ni** | **Zn** |
| Wavelength | 324.75 nm | 357.87 nm | 248.33 nm | 232.00 nm | 213.86 nm |
| Slit width | 0.70 nm | 0.70 nm | 0.20 nm | 0.20 nm | 0.70 nm |
| Lamp type | HCL | HCL | HCL | HCL | HCL |
| Lamp current | 15 | 25 | 30 | 25 | 15 |
| Flame | Air-Acetylene | Air-Acetylene | Air-Acetylene | Air-Acetylene | Air-Acetylene |
| Air flow (L/min) | 10.00 | 10.00 | 9.50 | 9.50 | 10.00 |
| Acetylene flow (L/min) | 2.50 | 3.30 | 3.00 | 2.60 | 2.50 |
| Signal type | BG-AA | BG-AA | BG-AA | BG-AA | BG-AA |
| Signal measurement | Time average | Time average | Time average | Time average | Time average |

BG-AA, Background corrected atomic absorption; HCL, Hollow cathode lamp

**Table 2BS**

Operating parameters for graphite furnace atomic absorption spectrometer (GFAAS)

| **Operating parameters** | **Analyte** | | | | |
| --- | --- | --- | --- | --- | --- |
|  | **Fe** | **Zn** | **Ni** | **Cd** | **Pb** |
| Wavelength | 248.33 nm | 213.86 nm | 232.0 nm | 228.80 nm | 283.31 nm |
| Slit width | 0.20 nm | 0.70 nm | 0.2 nm | 0.70 nm | 0.70 nm |
| Lamp type | HCL | HCL | HCL | EDL | EDL |
| Lamp current | 30 | 15 | 25 | 230 | 440 |
| Signal type | BG-AA | BG-AA | BG-AA | BG-AA | BG-AA |
| Signal measurement | Peak area | Peak area | Peak area | Peak area | Peak area |

BG-AA, Background corrected atomic absorption; HCL, Hollow cathode lamp; EDL, Electrodeless discharge lamp.

**Table 3AS**

Graphite furnace program for determination of Cd

| **Step** | **Temperature (°C)** | **Ramp time (sec)** | **Hold time (sec)** | **Argon Gas flow (mL/min)** | **Read** |
| --- | --- | --- | --- | --- | --- |
| - Drying | 110 | 1 | 30 | 250 | Off |
| - Pre-treatment | 130 | 20 | 20 | 250 | Off |
| - Pyrolysis | 850 | 5 | 20 | 250 | Off |
| - Atomization | 1650 | 0 | 5 | 0 | On |
| - Cleaning | 2600 | 1 | 5 | 250 | Off |

**Table 3BS**

Graphite furnace program for determination of Pb

| **Step** | **Temperature (°C)** | **Ramp time (sec)** | **Hold time (sec)** | **Argon Gas flow (mL/min)** | **Read** |
| --- | --- | --- | --- | --- | --- |
| - Drying | 110 | 1 | 30 | 250 | Off |
| - Pre-treatment | 130 | 15 | 20 | 250 | Off |
| - Pyrolysis | 700 | 10 | 20 | 250 | Off |
| - Atomization | 1800 | 0 | 5 | 0 | On |
| - Cleaning | 2600 | 1 | 5 | 250 | Off |

**Table 3CS**

Graphite furnace program for determination of Ni

| **Step** | **Temperature (°C)** | **Ramp time (sec)** | **Hold time (sec)** | **Argon Gas flow (mL/min)** | **Read** |
| --- | --- | --- | --- | --- | --- |
| - Drying | 110 | 1 | 30 | 250 | Off |
| - Pre-treatment | 130 | 15 | 25 | 250 | Off |
| - Pyrolysis | 1400 | 15 | 25 | 250 | Off |
| - Atomization | 2500 | 0 | 5 | 0 | On |
| - Cleaning | 2600 | 1 | 5 | 250 | Off |

**Table 3DS**

Graphite furnace program for determination of Cu

| **Step** | **Temperature (°C)** | **Ramp time (sec)** | **Hold time (sec)** | **Argon Gas flow (mL/min)** | **Read** |
| --- | --- | --- | --- | --- | --- |
| - Drying | 110 | 1 | 30 | 250 | Off |
| - Pre-treatment | 130 | 10 | 15 | 250 | Off |
| - Pyrolysis | 1000 | 15 | 15 | 250 | Off |
| - Atomization | 2300 | 0 | 5 | 0 | On |
| - Cleaning | 2600 | 1 | 5 | 250 | Off |

**Table 3ES**

Graphite furnace program for determination of Cr

| **Step** | **Temperature (°C)** | **Ramp time (sec)** | **Hold time (sec)** | **Argon Gas flow (mL/min)** | **Read** |
| --- | --- | --- | --- | --- | --- |
| - Drying | 110 | 1 | 30 | 250 | Off |
| - Pre-treatment | 130 | 15 | 20 | 250 | Off |
| - Pyrolysis | 1650 | 10 | 25 | 250 | Off |
| - Atomization | 2500 | 0 | 5 | 0 | On |
| - Cleaning | 2600 | 1 | 5 | 250 | Off |

**Table 4S**

Recovery percentage from spiked tea samples

| **Analyte** | **Concentration in control sample**  **(**mg kg^-1^**)** | **Amount added**  **(**mg kg^-1^**)** | **Concentration in**  **spiked sample**  **(**mg kg^-1^**)** | **Recovery (%)** |
| --- | --- | --- | --- | --- |
| Cu | 4.52±0.02 | 0.50 | 5.01±0.03 | 98.0 |
|  |  | 1.00 | 5.51±0.04 | 99.0 |
|  |  | 2.00 | 6.66±0.05 | 107.0 |
| Cr | 3.14±0.07 | 0.50 | 3.61±0.04 | 94.0 |
|  |  | 1.00 | 4.20±0.05 | 106.0 |
|  |  | 2.00 | 5.11±0.08 | 98.5 |
| Fe | 40.15±1.12 | 5.0 | 44.91±1.14 | 95.2 |
|  |  | 10.0 | 50.6±1.11 | 102.1 |
|  |  | 20.0 | 60.38±1.15 | 101.2 |
| Ni | 2.34±0.01 | 0.10 | 2.44±0.02 | 100.0 |
|  |  | 0.25 | 2.58±0.04 | 96.0 |
|  |  | 0.50 | 2.85±0.07 | 102.0 |
| Zn | 10.12±0.09 | 0.50 | 10.6±0.04 | 96.0 |
|  |  | 1.00 | 11.19±0.04 | 102.0 |
|  |  | 5.00 | 15.04±0.08 | 98.4 |
| Pb | 0.10±0.001 | 0.05 | 0.1451±0.04 | 90.2 |
|  |  | 0.10 | 0.1970±0.04 | 97.0 |
|  |  | 0.15 | 0.2470±0.08 | 98.0 |
| Cd | 0.02±0.001 | 0.01 | 0.0301±0.04 | 101.0 |
|  |  | 0.15 | 0.1589±0.04 | 92.6 |
|  |  | 0.02 | 0.0411±0.08 | 105.5 |

**Table 5S**

Hazard index (HI) values for both men and women from consumption of green tea

| **Cultivar** | **Men** | |  | **Women** | |
| --- | --- | --- | --- | --- | --- |
|  | **Orthodox green tea**×10^-2^ | **CTC green tea**×10^-2^ |  | **Orthodox green tea**×10^-2^ | **CTC green tea** ×10^-2^ |
| TV1 | 1.71±0.14 | 2.49±0.20 |  | 1.77±0.14 | 2.59±0.19 |
| TV9 | 2.70±0.17 | 3.70±0.36 |  | 2.81±0.18 | 3.84±0.38 |
| TV18 | 2.79±0.14 | 3.98±0.31 |  | 2.89±0.14 | 4.14±0.29 |
| TV20 | 1.86±0.12 | 2.70±0.20 |  | 1.94±0.12 | 2.81±0.21 |
| TV22 | 2.41±0.16 | 3.76±0.46 |  | 2.51±0.16 | 3.90±0.47 |
| TV23 | 2.72±0.18 | 3.85±0.49 |  | 2.82±0.19 | 4.00±0.51 |
| TV25 | 2.42±0.15 | 3.80±0.37 |  | 2.52±0.19 | 3.95±0.38 |
| RR17/144 | 1.71±0.15 | 2.67±0.15 |  | 1.83±0.16 | 2.77±0.16 |
| HV39 | 1.99±0.18 | 3.20±0.26 |  | 2.06±0.22 | 3.32±0.27 |
| Ging186 | 1.59±0.15 | 2.23±0.27 |  | 1.65±0.16 | 2.32±0.28 |
| 482/12 | 2.85±0.26 | 2.75±0.23 |  | 2.95±0.27 | 3.90±0.65 |

**Table 6S**

MANOVA test criteria and F approximations for the hypothesis of no overall cultivar effect for orthodox

| **Statistics** | **Value** | **F Value** | **Pr > F** |
| --- | --- | --- | --- |
| Wilks' Lambda | 0.0000000 | 79.76 | < 0.0001 |
| Pillai's Trace | 9.2577837 | 16.04 | < 0.0001 |
| Hotelling-Lawley Trace | 1814.0311372 | 99.81 | < 0.0001 |
| Roy's Greatest Root | 828.2068780 | 1064.84 | < 0.0001 |
| NOTE: F Statistic for Roy's Greatest Root is an upper bound. | | | |

**Table 7S**

MANOVA test criteria and F approximations for the hypothesis of no overall cultivar effect for CTC

| **Statistics** | **Value** | **F Value** | **Pr > F** |
| --- | --- | --- | --- |
| Wilks' Lambda | 0.0000000 | 61.09 | < 0.0001 |
| Pillai's Trace | 8.8424944 | 9.82 | < 0.0001 |
| Hotelling-Lawley Trace | 1828.6289024 | 100.61 | < 0.0001 |
| Roy's Greatest Root | 1013.7475548 | 1303.39 | < 0.0001 |
| NOTE: F Statistic for Roy's Greatest Root is an upper bound. | | | |

**Table 8S**

MANOVA test criteria and F approximations for the hypothesis of no overall cultivar effect for both the methods

| **Statistics** | **Value** | **F Value** | **Pr > F** |
| --- | --- | --- | --- |
| Wilks' Lambda | 0.00000000 | 41.87 | < 0.0001 |
| Pillai's Trace | 7.63004793 | 11.50 | < 0.0001 |
| Hotelling-Lawley Trace | 283.02832313 | 79.67 | < 0.0001 |
| Roy's Greatest Root | 133.07533577 | 475.27 | < 0.0001 |
| NOTE: F Statistic for Roy's Greatest Root is an upper bound. | | | |

**Table 9S**

MANOVA test criteria and exact F statistics for the hypothesis of no overall methods effect

| **Statistics** | **Value** | **F Value** | **Pr > F** |
| --- | --- | --- | --- |
| Wilks' Lambda | 0.02192298 | 130.66 | < 0.0001 |
| Pillai's Trace | 0.97807702 | 130.66 | < 0.0001 |
| Hotelling-Lawley Trace | 44.61424580 | 130.66 | < 0.0001 |
| Roy's Greatest Root | 44.61424580 | 130.66 | < 0.0001 |
